# Concentrated Foods

**Published:** 1885-07

**Authors:** 


### CONCENTRATED FOODS.

Medical men are now recognizing the value of malt extracts as foods in cases of deficient assimilation. That their use is extending may be taken for granted by the numbers of exhibitors of concentrated foods in the exhibition at South Kensington, last year. Important improvements have recently been made in the manufacture of malt extracts, which are now prescribed in a variety of forms. One of the most effective combinations in dyspepsia, cholera infantum, and all diseases resulting from imperfect nutrition is maltine, with pepsine and pancreatine, containing, as it does, three of the all-important digestive agents, diastase being one of the constituents of maltine. Dyspepsia, in most cases, will be found to yield to the medicinal properties of this combination, while the system is invigorated by its nutri tive qualities. It will be found a useful remedy also for constipation and chronic diarrhœa, resulting from mal-nutrition. Not only is maltine of itself of great value in certain cases, but it may be combined with the most valuable alteratives known, such as iodides, bromides, and chlorides, and is found to be a remedy of high value in all depraved conditions of the blood. The maltine manufactured by the Maltine Manufacturing Company, of New York, bears a high name, and this has been still further emphasized by the award of the Gold Medal of the Health Exhibition, London, for their malt extract known as maltine (malted wheat, barley and oats), the only preparation composed of these three cereals. Professor Charles R. C. Tichborne, after an examination of the principal unfermented extracts of malt in the market, finds that maltine is the richest in two of the most important ingredients in these foods, namely, the phosphates or bone-formers, and that peculiar farinaceous digestive agent called diastase. Maltine may be said to consist of about eighty per cent. of pure food in its most concentrated and assimilable form. This eighty per cent. may be divided as follows: Five and a half per cent. of flesh-formers; seven per cent. of heat-givers; two per cent. of bone-formers; add to this the diastase, which imparts to it the curious power of digesting all farinaceous food outside itself, and we have in maltine a most valuable adjunct to our invalid diet. In respect to the diastase, maltine seems remarkably energetic, and at the temperature of the human body one part liquified "twenty parts of starch in two minutes," and had completely changed or digested that body in about an hour. Maltine possesses all the characteristics of a cereal extract, as prepared from the grain, and there can be no question about the genuineness of this preparation. It is only necessary to consult any work upon dietetics to see that there is considerable difference in the composition of the various grain crops. By combining these three important substances, barley, oats and wheat, a food is obtained which represents the average composition of the three cereals, and that food, already digested for use, a condition of immense value to the physician in those special cases where the digestive functions are impaired.—*Midland Medical Journal, Leicester and London, Jan. 1, 1885.*

